# Exosomal KRAS mutation promotes the formation of tumor-associated neutrophil extracellular traps and causes deterioration of colorectal cancer by inducing IL-8 expression

**DOI:** 10.1186/s12964-020-0517-1

**Published:** 2020-03-30

**Authors:** Anquan Shang, Chenzheng Gu, Chen Zhou, Yibao Yang, Chen Chen, Bingjie Zeng, Junlu Wu, Wenying Lu, Weiwei Wang, Zujun Sun, Dong Li

**Affiliations:** 1 Department of Laboratory Medicine, Shanghai Tongji Hospital, Tongji University School of Medicine, No. 389, Xincun Road, Shanghai, 200065 People’s Republic of China; 2 Department of Pathology, The Sixth People’s Hospital of Yancheng City, Yancheng, 224001 People’s Republic of China

**Keywords:** Colorectal cancer, KRAS mutant, IL-8, Exosome, Neutrophil extracellular trap, Neutrophil recruitment

## Abstract

**Background:**

Colorectal cancer (CRC) remains one of the leading causes of cancer-related death. The current study aimed to elucidate the mechanism by which exosomes carrying KRAS mutant contribute to neutrophil recruitment as well as the formation of the neutrophil extracellular trap (NET) in CRC.

**Methods:**

APC-WT and APC-KRAS^G12D^ mouse models were initially developed. Peripheral blood, spleen, bone marrow (BM) and mesenteric lymph nodes (mLN) were isolated to detect neutrophil content. Then, APC-WT and APC-KRAS^G12D^ mice were injected with exosomes isolated from APC-WT and APC-KRAS^G12D^ mice. The ratio of neutrophils, NETs formation and IL-8 protein content were subsequently quantified in colon tissues. DKs-8 (wild type) and DKO-1 (KRAS mutant) cells were employed for in vitro experimentation. Then, DKs-8 cells were cultured with exosome-treated PMA stimulated neutrophil-forming NETs culture medium, with cell viability, invasion, migration, and adhesion evaluated.

**Results:**

Compared with APC-WT mice, the numbers of polyps and neutrophils in the peripheral blood, spleen and mLNs were increased in APC-KRAS^G12D^ mice, accompanied with increased NET formation, IL-8 expression and exosomes. Meanwhile, IL-8 upregulation, neutrophil recruitment and NET formation were observed in the mice injected with exosomes derived from APC-KRAS^G12D^. The in vitro investigation results revealed that more NETs were formed in the presence of DKO-1-Exos, which were inhibited by DNAse. In addition, DKs-8- and DKO-1 cells-derived exosomes could adhere to NETs under static conditions in vitro. Exosomal KRAS mutants were noted to exert stimulatory effects on the IL-8 production and NET formation to promote the growth of CRC cells.

**Conclusion:**

The results provide evidence suggesting that exosomes may transfer mutant KRAS to recipient cells and trigger increases in IL-8 production, neutrophil recruitment and formation of NETs, eventually leading to the deterioration of CRC.

## Background

Colorectal cancer (CRC), also known as colon cancer, is a malignancy that predominately afflicts the terminal region of large intestine. Statistically speaking, CRC ranks as the third most common cancer and the fourth most common cause of cancer related deaths worldwide, with over one million new cases and around 600 thousand deaths per year [1, 2]. CRC gives rise to bowl pain as well as regional inflammation. Patients with advanced CRC typically suffer from extensive metastasis of CRC. The 5-year survival rate for patients at primary stage is over 90%, however, that for patients with distant metastasis is just about 10% [1, 3]. Although early prognosis of CRC greatly improves and more patients survive, effective treatments of CRC often require a combination of resection, chemotherapy or radiation therapy according to metastatic status of CRC cells. The risk factors of CRC are majorly comprised of an unhealthy lifestyle and advancing age, while genetic factors account for approximately 15 to 30% [4]. Inherited genetic defects such as gene alterations typically include APC, MSH2, KRAS, PTEN and P53 involving the WNT pathway, EGFR signaling pathway and TGF-β signaling pathway [5].

KRAS protein is a member of the RAS family containing KRAS, HRAS and NRAS, which are small G proteins that regulate membrane signaling transition and cellular proliferation [6]. KRAS mutations have been implicated in approximately in nearly 40% CRC cases, as well as in many other types of human cancers such as lung cancer, breast cancer, prostate cancer and etc. [5]. Hence, various investigations have identified it as clinically prognostic and predictive marker in CRC [7, 8]. Existing literature has demonstrated the potential of KRAS to regulate epithelial transition and proliferation of cancer cells through Wnt signaling pathway and MAPK signaling pathway, respectively [9], which help cure CRC patients by targeted therapy. However, recent studies have also implied that cancer microenvironment in tissue is strongly involved with cancer progression besides oncogenic alteration [10]. It is believed that regional inflammatory response occurs when tumor cells emerge in tissue, so does CRC [11]. One typical example is that the colitis-associated cancer (CAC), one of the subtypes of CRC, is strongly associated with inflammatory bowel disease (IBD) [12]. Evidence has been previously presented indicating that cancer cells can secrete exosomes to regulate tissue microenvironment. However, the mechanism by which cancer cells escape from immunosurveillance and abduct some immune cells to promote proliferation of cancer cells remains unclear. Thus, during the current study we established a KRAS mutation mouse model with the objective of elucidating the relationship of interlukin-8 (IL-8) and neutrophil extracellular traps (NETs) in KRAS-mutant CRC.

## Methods and materials

### Ethics statement

The study protocol was approved by the Animal Ethics Committee of Shanghai Tongji Hospital, Tongji University School of Medicine. Animal use and experiments were carried out in strict accordance with the procedures approved by the National Cancer Institute Animal Care and Use Committee (ACUC).

### Establishment of animal model

We utilized a genetically engineered mouse model where a localized infection of colonic tissue with adenovirus expressing Cre recombinase results in a tumor driven by either loss of both copies of APC with wild-type Kras (APC-WT) or a tumor driven by loss of APC with activated Kras harboring a gain in function mutation Kras^G12D^ (APC-KRAS^G12D^) using the methods described previously [13]. APC-KRAS^G12D^ mice are produced by hybridization of the homozygous APC allele with a heterozygous LSL-Kras^G12D/+^ allele. The APC/Kras^mut^ mice were then hybridized to restore the homozygous APC allele and the heterozygous LSL-Kras. At the age of 7 weeks, APC and APC/KRAS mice received an intraperitoneal injection of tamoxifen (20 mg/kg, Sigma, Cat. #T5648) to activate stem cell-specific Cre. The activated Cre could induce APC loss and KRAS G12D allele activation. On average, more than 100 polyps were observed in the distal ileum (ileum) of the genetically engineered mouse 10 weeks after induction of gene mutation. All the APC-WT and *APC-KRAS*^*G12D*^ mice were euthanized at 8th, 12th, 16th, and 20th weeks (eight mice each time), and the number of polyps was counted.

### Neutrophil isolation and sorting

Neutrophils were isolated from peripheral blood, spleen, bone marrow (BM) and mesenteric lymph nodes (mLN) of APC-WT and APC-KRAS^G12D^ mice. Neutrophils were sorted on a BD-Aria-Plus high-speed sorter after incubation with APC-conjugated anti-mouseLy6G antibody (BD Biosciences, San Jose, California, USA) and APC-Cy7 CD11b (BD Biosciences, San Jose, California, USA).

### NETs quantification in vivo

To quantify the NETs in vivo, a capture enzyme-linked immunosorbent assay (ELISA) was performed to identify myeloperoxidase (MPO)-DNA complexes, as MPO was identified as a prominent constituent of NETS. The MPO-DNA complex was detected according to the instructions of the Mouse MPO ELISA kit (HK210–01; Hycult Biotechnology BV, Uden, the Netherlands). Briefly, 100 μL of the sample was added to the culture well, and incubated with 300 μL peroxidase-labeled anti-DNA mAb (Cat. No: 11774424001; Cell Death Detection ELISA PLUS, Roche Diagnostics GmbH, Mannheim, Germany) for 1 h. After that, the absorbance was measured. Values for soluble NET formation are expressed as percentage increase in absorbance above control.

### Isolation of exosomes from plasma samples and cell culture supernatants

The plasma samples from APC-WT and APC-KRAS^G12D^ mice were centrifuged at 1000 g for 10 min. The harvested supernatant was further diluted in phosphate-buffered saline (PBS) and ultra-centrifuged at 100,000 g for 1 h at 4 °C using a Beckman Coulter Optima LE-80 K ultracentrifuge (Beckman Coulter, Fullerton, CA, USA). The pellet was resuspended in ice cold PBS, filtered with a 0.2 μm pore size filter, and preserved at − 20 °C.

Exosomes were isolated from the conditioned medium of DKs-8 and DKO-1 cells, respectively. The fetal bovine serum (FBS) was ultra-centrifuged at 100,000 g for 8 h in advance to remove the exosomes from the serum. Cells were cultured in Dulbecco’s Modified Eagle Medium (DMEM) supplemented with 10% FBS until 80% cell confluence. After the cells were cultured in the medium containing 10% exosome-free FBS for 48 h, the cells were collected and centrifuged at 300 g for 10 min to remove cell debris, after which the supernatant was filtered through a 0.22 um polyethersulfone filter (Nalgene, Rochester, NY, USA) and subsequently concentrated using a 100,000 molecular weight cut-off Amicon Ultra centrifugal filter (Millipore, Billerica, MA, USA) and centrifuged at high speed of 150,000 g for 2 h to obtain the exosomes. Protein concentration of the exosomal preparations was determined using a microBCA protein assay kit (Pierce, Rockford, IL, USA), and the number of exosomes per microgram of protein was determined by nanoparticle tracking analysis (NanoSight, Wiltshire, UK).

### Exosomes injection in vivo

A total of 5 mg APC-WT and APC-KRAS^G12D^ serum exosomes were intravenously injected into the tail vein of mice every 3 days. Exosomes used in these experiments were previously labeled with membrane fluorescent tracer PKH67 (Sigma-Aldrich). The labeled exosomes were washed, collected by ultracentrifugation, and resuspended in RPMI 1640 medium. Exosome-PKH67 and neutrophils (CD11b^+^ Ly6G^+^) were evaluated by flow cytometric analysis performed on a FACS LSR Fortessa flow cytometer with FACSDiva software (BD Biosciences, San Jose, CA, USA).

### Cell culture

DKs-8 (WT allele), DKO-1 (KRAS mutant) cells were cultured in serum-containing DMEM (Mediatech, Manassas, VA, USA). Human peripheral blood neutrophils (PMN) were prepared as described elsewhere [14].

### Three-dimensional cell culture

Three layers of collagen were used in a 48-well cell culture dish for cell culture in a three-dimensional collagen gel matrix. Neutrophils were trypsinized, syringed, and resuspended in serum-free DMEM at a concentration of 5 × 10^5^ cells/mL before being plated in collagen-coated dish. The upper and lower layers contained 150 μL/well of PureCol collagen (Advanced Biomatrix, San Diego, CA) which were diluted to 2 mg/ml in serum-free DMEM. The intermediate layer consisted of 2 mg/mL collagen in serum-free DMEM (150 μL/well) and 5 × 10^3^ neutrophils. Serum-free medium or serum-free medium supplemented with 50 g of DKs-8- or DKO-1-derived exosomes. The medium was renewed twice a week, and the neutrophils was cultured for 2 weeks. The experiment was repeated independently three times.

### Detection of peptides via LC-multiple reaction monitoring (LC-MRM)

DKs-8, DKO-1 cells and PMN were grown to 80% confluence. After serum starvation overnight, one million cells were cocultured with 100 g of the indicated exosomes or mock-treated for 1 h under constant rotation at 37 °C. Cells were then pelleted and rinsed three times with ice-cold PBS, and the detection of peptides was conducted as described previously [15]. The mobile phase consisted of 0.1% formic acid solution (A) or 90% acetonitrile (B). An 80 min gradient was performed with a 15 min washing period (100% A). Following that, the gradient was increased to 60% B for 43 min treatment, followed by an increase to 95% B for 49 min treatment. At last, the cells were allowed to stand for 11 min before the gradient returning to 97% A. Transitions for each peptide (WT KAS LVVVGAGGVGK, KRAS G13D LVVVGAGDVGK) were selected using the Skyline software package.

### In vitro NET formation

Neutrophils were incubated with tumor exosomes, plated on coated plates for 1 h, and then treated with Phorbol-12-myristate-13-acetate (PMA, 100 nM, Sigma-Aldrich) at 37 °C 5% CO_2_ for 4 h. Equivalent cells were also lysed with 0.1% Triton X-100 (Union Carbide Chemicals, Cleveland, OH, USA) or frozen as a control for the total DNA. Cells were incubated at 37 °C for 2–4 h. The membrane-impermeable DNA-binding dye, Sytox Green (Invitrogen) was added (0.1 μM) to bind extracellular DNA, and fluorescence was quantified using a fluorescence spectrophotometer (Thermo Fisher Scientific, Waltham, MA, USA).

### Interaction of exosomes with NETs under static conditions

Neutrophils (1 × 10^5^) were seeded onto 13 mm coverslips (Glasscyto) and treated with 50 nM Phorbol-12-myristate-13-acetate (PMA, 100 nM, Sigma-Aldrich), a potent inducer of NETosis at 37 °C, 5% CO_2_ for 4 h. The neutrophils were then cocultured with 1 μg of exosomes which were labeled with 3 mM DiD in advance. The neutrophils were fixed with 500 uL of 4% paraformaldehyde for 10 min, then washed 3 times with PBS and incubated for 10 min with blocking solution (PBS, 10% FBS, 5 mg/ml BSA). The samples were subsequently stained with Hoechst 33342 (1:1000) for 2 h. Images were captured with a confocal microscope (LEICA DMI 4000 with laser system TCS SPE) and analyzed using Fiji Image J software.

### Adhesion, migration and invasion assays

For the adhesion assays, 5 × 10^5^ neutrophils were encapsulated in 24-well plates for 1 h at 37 °C in 5% CO_2_. DKs-8 cells were labeled with CFSE (Molecular Probes) for 10 min, and then 1 × 10^5^ DKs-8 cells were added to the wells containing 100 nM PMA neutrophils, DKs-8 exosomes (DKs-8 exos); DKO-1 exosomes (DKO-1 exos); DKs-8 exos and 1000 U DNAse, DKO-1 exos and IL-8 or DKO-1 exos and anti-IL-8). Wells without PMA were used as controls. After incubation for 4 h at 37 °C in 5% CO_2_, wells were fixed with 4% paraformaldehyde. Cell adhesion was evaluated as the number of CFSE-labeled DKs-8 cells in 4 random high power field at × 20 using Olympus Floview 500 microscope.

Cell migration and invasion were tested using 6.5-mm Costar Transwell chambers with 8-μm pores (Corning, NY, USA). The plate coated with 50 μL Matrigel (YB356234, Shanghai Yubo Biological Technology, Shanghai, China) diluted by serum-free medium at 4 °C was then placed into the upper chamber of the Transwell apparatus. Then, 200 μL of cell suspension in medium containing 20% FBS was added to each well of the upper chamber and 800 μL of conditional medium containing 20% FBS was added to the lower chamber. Tumor cell suspension in the upper chamber were treated with neutrophil media containing DKs-8 exos + PMA-stimulated neutrophils, DKO-1 exos + PMA-stimulated neutrophils, DKO-1 exos + PMA-stimulated neutrophil media and 1000 U of DNAse, DKO-1 exos + PMA-stimulated neutrophil and 10 μg/ml IL-8 neutralizing antibodies, DKO-1 exos + PMA-stimulated neutrophil and 10 μg/ml anti-IL-8, or culture media alone (control). After incubation for 24 h at 37 °C, transwell plate was immersed in formaldehyde for 10 min, and then stained with 0.1% crystal violet for observation and cells were counted under an inverted microscope.

### Cell counting kit-8 (CCK-8)

Cell viability was detected by CCK-8 kit (Dojindo Laboratories, Kumamoto, Japan). DKs-8 cells (1 × 10^3^ cells/well) were seeded onto 96-well plates and incubated for 48 h before CCK-8 detection. Ten μL of CCK-8 test solution was added into each well for incubation at 37 °C. At each time point (12, 24, 48 h), the optical density (OD) at 450 nm wavelength was read with a microplate reader.

### Elisa

The IL-8 concentration in the cell culture supernatant (1 × 10^6^ cells/mL) and the concentrations of TNF-α, IL-6, IL-8, and IL-17 in the serum were measured according to the ELISA kit instructions (R&D Systems, Minneapolis, MN, USA). The concentration of G-CSF in mouse serum was measured using a Mouse G-CSF ELISA Kit (ab197743, Abcam Inc., Cambridge, UK).

### RNA extraction and quantification

Total RNA was extracted using miRNeasy Mini Kit (217,004, QIAGEN, Germany). The total RNA concentration and purity were determined on a nanodrop2000 micro-ultraviolet spectrophotometer (1011 U, nanodrop, USA). The RNA was reversely transcribed into cDNA using TaqMan MicroRNA Assays Reverse Transcription Primer kit (4,427,975, Applied Biosystems, USA). IL-8 primers and probes were purchased from Applied Biosystems (Test ID: Hs00174103_m1). The following primers and probe sequences and concentrations were used for human β-actin control detection: 5’Primer: GCCACCCCACTTCTCTCTAAGG at 50 nM, 3’Primer: GGGCACGAAGGCTCATCATTC at 300 nM, and TaqMan Probe: VIC-CCCAGTCCTCTCCCAAGTCCACACAGG-TAMRA at 50 nM. Real-time quantitative polymerase chain reaction (qPCR) was performed using ABI 7500 quantitative PCR instrument (Applied Biosystems, Foster City, CA, USA). The final data were analyzed using the 2^-ΔΔCt^ method and experiments were repeated independently three times.

### Western blot analysis

Total protein was extracted and quantified using 2D Quant kit (GE Healthcare, Amersham Biosciences, Uppsala, Sweden). Then, 20 μg protein samples was separated by sodium dodecyl sulfate-polyacrylamide gel electrophoresis (SDS-PAGE) and transferred onto membrane. After blocking, the membrane was incubated with primary antibodies overnight at 4 °C. KRAS (ab180772, 1:500), CD63 (ab68418, 1:1000), CD9 (ab223052, 1:1000), CD81 (ab155760, 1:1000), and Calnexin (ab22595, 1:1000) were purchased from Abcam (Cambridge, UK). After intensive washing, the membrane was further incubated with IgG goat anti-rabbit secondary antibody (1:1000, A21020, Abbkine, Scientific Co., Ltd., Wuhan, China) for 1 h at 37 °C, and was then developed by enhanced chemiluminescence (ECL) reagent. GAPDH worked as an internal control. The experiment was repeated independently three times.

### Statistical analysis

SPSS 21.0 (IBM Corporation, San Jose, CA, USA) was used for statistical analysis. The measurement data with normal distribution was expressed as mean ± standard deviation. The unpaired *t*-test was used to compare the two independent groups that data conformed to the normal distribution and equal variance. Data comparisons between groups were performed using one-way analysis of variance (ANOVA), followed by Tukey’s post hoc test. Data analysis at different time points was performed by repeated measurement ANOVA with Bonferroni post hoc test. The difference was regarded as statistically significant when *p* < 0.05.

## Results

### Neutrophil accumulation involves in intestinal tumor deterioration in KRAS-mutated CRC

**Fig. 1:**
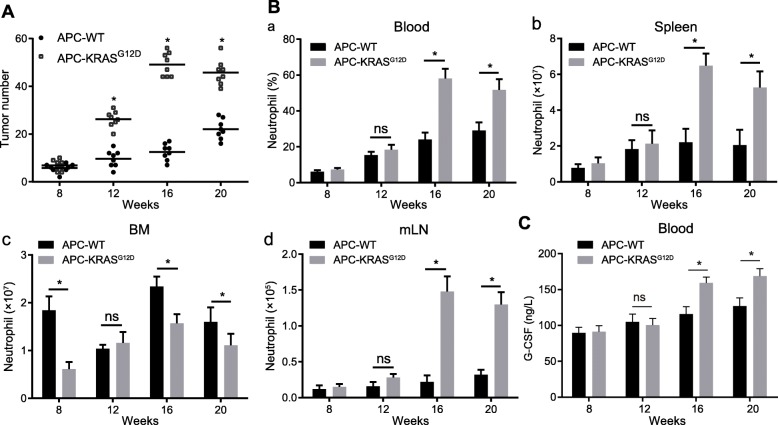
Neutrophil accumulation involves in intestinal tumor deterioration in KRAS-mutated CRC; A, APC-WT and APC-KRAS^G12D^ mice at 8th, 12th, 16th and 20th weeks were euthanized. Tumor number was evaluated by counting single polyps in small colons. B, The number of neutrophils counted using flow cytometry, Single-cell suspensions were prepared from the peripheral blood (**a**), spleen (**b**), BM(**c**) and mLN (**d**) of APC-WT and APC-KRAS^G12D^ mice euthanized at indicated ages. The graphs show total numbers or frequencies of CD45.2 þ Ly6G þ CD11b þ neutrophils in respective organs. C, The content of G-CSF in the serum of APC-WT and APC-KRAS^G12D^ mice. * *p* < 0.05, *n* = 8. The measurement data was expressed as mean ± standard deviation. Data analysis at different time points was performed by repeated measurement ANOVA, follows by Bonferroni post hoc test

In order to assess the role of KRAS mutations in intestinal tumorigenesis, we constructed a APC-KRAS^G12D^ mouse model. The number of intestinal polyps in APC-WT and APC-KRAS^G12D^ mice was counted at different time points. As depicted in Fig. 1a, the number of polyps in APC-KRAS^G12D^ mice was significantly higher than in APC-WT mice from 12 weeks (*p < 0.05). Next, in order to understand whether the development of KRAS-mutated mouse intestinal tumors is associated with increased neutrophils, we examined the number of neutrophils in peripheral blood, spleen, BM and mLN of APC-WT and APC-KRAS^G12D^ mice at different time points. As illustrated in Fig. 1b (a-d), at 8th weeks, the numbers of neutrophils in blood, spleen, and mLN were not significantly different between APC-WT and APC-KRAS^G12D^ mice. In contrast, the results depicted in Fig. 1b (c) displayed that the neutrophils were significantly decreased in the bone marrow of KRAS-mutated APC-KRAS^G12D^ mice. At 12th weeks, the number of neutrophils was increased in the spleen, blood and mLN of APC-KRAS^G12D^ mice, but no significant differences was identified. At 16th weeks, the number of neutrophils reached a peak, and significant increases persisted throughout the subsequent period until 20 weeks. The results of ELISA displayed that the level of G-CSF was elevated in the serum of APC-KRAS^G12D^ mice than in APC-WT mice (Fig. 1c). G-CSF exerted promoting effects on the differentiation and proliferation of neutrophils [16], which were consistent with the number of neutrophils in Fig. 1b.

### Exosomes derived from mice with KRAS mutant-CRC mediate IL-8 activation and NET formation

**Fig. 2:**
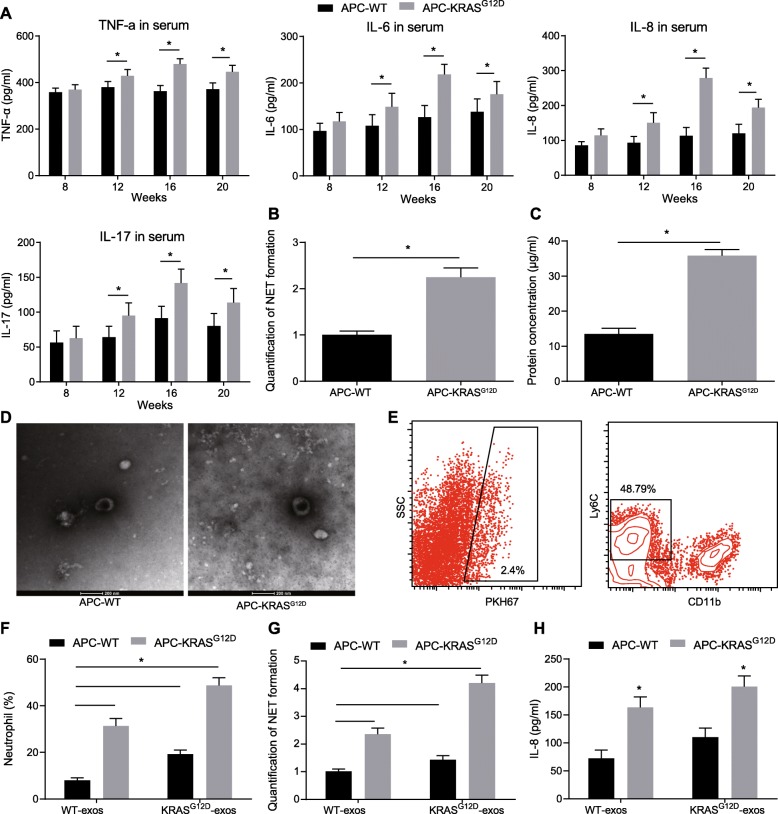
Exosomes derived from mice with KRAS mutant-CRC mediate IL-8 activation and NET formation; **a**, protein concentrations of TNF-α, IL-6, IL-8, IL-17 in the serum of APC-WT and APC-KRAS^G12D^ mice detected by ELISA; **b**, NETs in mice, detected by capture ELISA. Values for soluble NET formation are expressed as percentage increase in absorbance above control. Fold increase vs. APC-WT; **c**, Content of exosomes in serum of APC-WT and APC-KRAS^G12D^ mice determined by microBCA protein assay kit at 16th week; **d**, (Left) Electron microscopy images of APC^−^WT and APC-KRAS^G12D^ exosomes, Scale bars = 200 nm; (Right) Size (diameter) distribution of APC^−^WT and APC-KRAS^G12D^ exosomes; **e**, flow cytometry detection of neutrophils binding to PKH67-labeled KRAS mutant-derived exosomes; **f**, Ratio of neutrophils in colon tissue after administration of exosome of APC-WT and APC-KRAS^G12D^ mice; **g**, Quantification of NET formation after exosome administration of APC-WT and APC-KRAS^G12D^ mice; **h**, IL-8 content in serum of APC-WT and APC-KRAS^G12D^ after administration of KRAS mutant exosomes. * *p* < 0.05, *n* = 8; The measurement data was expressed as mean ± standard deviation. An unpaired t test was used to compare the two groups. Data analysis at different time points was performed by repeated measurement ANOVA with Bonferroni post hoc test

It has been reported that compared with normal people, neutrophil trap formation and abnormal upregulation of IL-8 are near the lesions of CRC patients [17], and IL-8 can directly induce the production of NETs [18]. We examined the levels of TNF-α, IL-6, IL-8, and IL-17 in serum from APC-WT and APC-KRAS^G12D^ mice at different time points. The results showed (Fig. 2a) that compared with APC-WT mice, the levels of TNF-α, IL-6, IL-8, and IL-17 in the serum of APC-KRAS^G12D^ mice were significantly increased at 12th weeks, which reached the peak values at 16th weeks, and the IL-8 upregulation was more apparent among them. Moreover, our results demonstrated that the formation of NETs in APC-KRAS^G12D^ mice significantly was enhanced at 16th weeks (Fig. 2b).

We hypothesized that APC-KRAS^G12D^-derived exosomes may stimulate the activation of CRC cell chemokine IL-8, thereby promoting the formation of NETs. We first isolated exosomes in the serum of APC-WT and APC-KRAS^G12D^ mice and subsequently detected the content of exosomes in the serum of APC-WT and APC-KRAS^G12D^ mice. Exosomes in the serum of APC-KRAS^G12D^ mice were significantly enriched relative to that in the serum of APC-WT mice (Fig. 2c-d). Next, to investigate the role of APC-KRAS^G12D^-derived exosomes in the formation of NETs, the exosomes isolated from APC-WT and APC-KRAS^G12D^ mice were injected into APC-WT and APC-KRAS^G12D^ mice, and then whether the exogenously administered exosomes could be enriched in colon tissue and internalized by tumor cells was assessed. The positive cells in the PKH67-labeled exosomes were also assessed following administration. Flow cytometric results exhibited that 48.79% of exosome-positive cells were neutrophil cells (Fig. 2e). We subsequently investigated the role of APC-KRAS^G12D^-derived exosomes in the formation of NETs and recruitment of neutrophils in colon tissues. The results obtained demonstrated the content of neutrophils and the production of NETs were significantly increased in the APC-WT mice injected with APC-KRAS^G12D^-derived exosomes, and the similar increases were observed in APC-KRAS^G12D^ mice after treatment of APC-KRAS^G12D^-derived exosomes (Fig. 2f, g). Furthermore, we tested the IL-8 protein levels, as shown in Fig. 2h, IL-8 protein levels were also significantly elevated. The aforementioned data indicated that in vivo KRAS mutant exosomes could activate IL-8 expression, and induce neutrophil recruitment and NET formation.

### CRC cells transfer mutant KRAS to neutrophils via exosomes

**Fig. 3:**
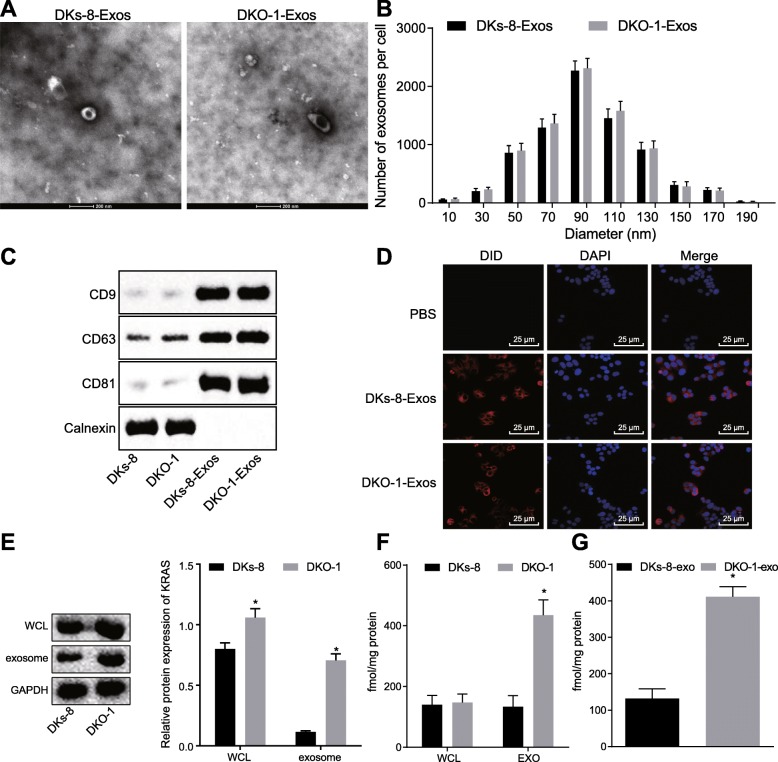
CRC cells transfer KRAS mutants to neutrophils via exosomes; **a**, DKs-8- and DKO-1-derived exosomes viewed under electron microscope; **b**, Size (diameter) distribution of DKs-8- and DKO-1-derived exosomes; **c**, The expression of exosome surface marker measured by Western blot analysis; **d**, Exosomes can be internalized by neutrophils revealed by Confocal microscopy (400×); **e**, The expression of KRAS in DKs-8 and DKO-1 cells (WCL) and their derived exosomes (EXO) measured by Western blot analysis; **f**, The presence of KRAS in DKs-8 and DKO-1 cells and their derived exosomes. Proteins from DKs-8 and DKO-1 cells (WCL) and their derived exosomes (EXO) were resolved via SDS-PAGE and subjected to targeted LC-MRM analysis for WT (LVVVGAGGVGK) and mutant (LVVVGAGDVGK) (G13D) KRAS peptides; **g**, mutant (G13D) KRAS in neutrophils LC-MRM detected by LC-MRM. * *p* < 0.05. The measurement data was expressed as mean ± standard deviation. An unpaired t test was used to compare the two groups. Data analysis at different time points was performed by repeated measurement ANOVA with Bonferroni post hoc test. The experiment repeated three times

To examine whether exosomes transfer mutant KRAS to neutrophils, we selected two CRC cell lines, the wild type allele DKs-8 cells and the KRAS mutant DKO-1 cells. Exosomes were isolated from DKs-8 and DKO-1 cells. The size analysis by transmission electron microscopy (Fig. 3a) showed that exosomes secreted by DKs-8 cells and DKO-1 cells had round membrane-like structures with a diameter of about 80–120 nm (Fig. 3b). The western blot analysis showed that both DKs-8- and DKO-1-derived exosomes (DKs-8-Exos and DKO-1-Exos) were positive for specific exosomal markers CD63, CD9 and CD81 but negative for the endoplasmic reticulum molecular chaperone protein Calnexin (Fig. 3c). To test whether exosome could be internalized by neutrophils, neutrophils were cocultured with DiD-labeled exosomes. Confocal microscopic images confirmed that neutrophils could take up DiD-labeled exosomes (Fig. 3d).

We next assessed whether mutant KRAS can be transferred from DKO-1 cells to neutrophils by exosomes, the expression of KRAS in the DKs-8-Exos and DKO-1-Exos were determined by western blot assay, which showed that the KRAS protein was enriched in the exosomes secreted from KRAS-mutated DKO-1 cells relative to the exosomes secreted from WT DKs-8 cells (Fig. 3e). However, this method could only detect the total KRAS protein but failed to distinguish between the mutant and wild-type KRAS proteins. Therefore, in order to distinguish between WT and KRAS mutants, we used LC-MRM. We designed KRAS WT (LVVVGAGGVGK) and mutant (LVVVGAGDVGK) (G13D) specific peptides and subsequently determined the expression of WT and mutant KRAS in DKs-8 and DKO-1 cell lines. In general, KRAS polypeptides are more abundant in exosomes than in cells. No significant difference was observed in the contents of WT peptide and G13D mutant peptide in the cells, while the content of G13D mutant peptide in DKO-1-Exos was higher than that of WT peptide (Fig. 3f).

After confirming the presence of the mutant KRAS in the DKO-1-Exos, we subsequently set out to verify that whether the exosomes could communicate with cells by cellular horizontal transfer of proteins to the recipient cells, and we examined whether exosomes transferred mutant KRAS to neutrophils. Neutrophils were cocultured with DKs-8-Exos or DKO-1-Exos for 1 h, and LC-MRM was used to detect G13D and WT polypeptides in neutrophils. Following incubation with DKO-1-Exos, G13D mutant peptide was detected in neutrophils (Fig. 3g). These results indicated that the mutant KRAS was transferred from DKO-1 cells to neutrophils by exosomes.

### Exosomes derived from DKO-1 cells induce NETs formation

**Fig. 4:**
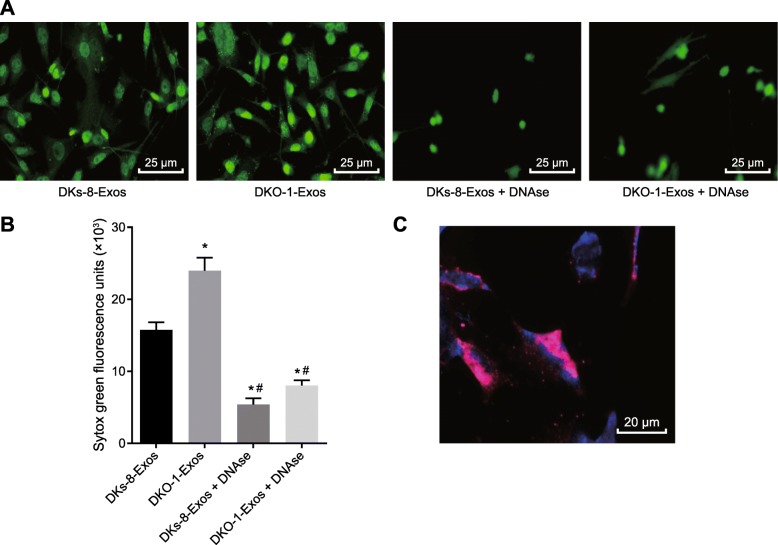
Exosomes derived from DKO-1 cells induce NETs formation; **a**, NETs formation after DKs-8-Exos and DKO-1-Exos were co-cultured with neutrophils (400×); **b**, fluorescence quantitative assessment of extracellular DNA fluorescence staining. Statistical comparisons were performed using the Mann Whitney U test; **c**, the interaction of DKO-1 derived exosomes with NETs observed by confocal microscopy (500×). The exosomes are labeled DiD (red) and the NETs are labeled Hoechst 33342 (blue). Neutrophils were isolated and induced to form NETs by stimulation with PMA for 4 h and then incubated with DKO-1-Exos exosomes. * *p* < 0.05 compared to DKs-8-EXos, and # *p* < 0.05 compared to DKO-1 Exos. The measurement data was expressed as mean ± standard deviation. Data comparisons between groups were performed using one-way analysis of variance, followed by Tukey’s post hoc test. The experiment repeated three times

The DKs-8-Exos or DKO-1-Exos were cocultured with neutrophils, and the formation of NETs in their derived exosomes was detected. Compared with DKs-8-Exos, more NETs were formed in DKO-1-Exos, while DNAse treatment was observed to suppress the formation of NETs (Fig. 4a, b). In addition, we identified that DKs-8-Exos or DKO-1-Exos could adhere to NETs under static conditions in vitro (Fig. 4c). These results indicated that neutrophils are capable of internalizing DKO-1-Exos and induce the formation of NETs.

### Mutant KRAS exosomes induce formation of NETs through upregulation of IL-8

**Fig. 5:**
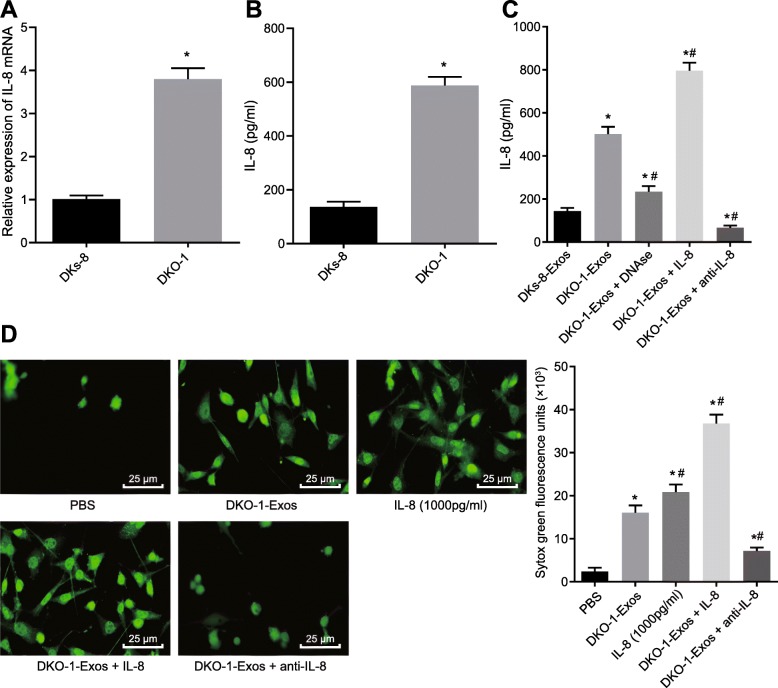
Mutant KRAS exosomes induce formation of NETs through upregulation of IL-8; **a**, mRNA expression of IL-8 in DKs-8 and DKO-1 cells determined by RT-qPCR, * *p* < 0.05 compared with DKs-8; **b**, IL-8 protein levels in culture medium after 48 h of DKs-8 and DKO-1 cell culture, analyzed by ELISA, * *p* < 0.05 compared with DKs-8; **c**, IL-8 protein levels in culture medium after alternative treatment* *p* < 0.05 compared with DKs-8 Exos, # *p* < 0.05 compared with DKO-1 Exos; **d**, NETs formation and fluorescence quantitative assessment of extracellular DNA fluorescence staining (400×). * *p* < 0.05 compared with PBS, # *p* < 0.05 compared to DKO-1 Exos. The measurement data was expressed as mean ± standard deviation. Data comparisons between groups were performed using one-way analysis of variance, followed by Tukey’s post hoc test. The experiment repeated three times

In order to assess the relationship between IL-8 expression and KRAS mutations in CRC cells in vitro, we initially examined the basal mRNA and protein expression of IL-8 mRNA in DKs-8 and DKO-1 cells by RT-qPCR and ELISA. The results showed that mRNA and protein levels of IL-8 in DKO-1 cells carrying the KRAS mutation were notably higher than those in DKs-8 cells with WT KRAS (Fig. 5a, b). To investigate the relationship between mutant KRAS exosomes-mediated formation of NETs and IL-8 expression, we examined the expression of IL-8 after co-culture of neutrophils with DKs-8-Exos or DKO-1-Exos in the presence of PMA. The results of ELISA exhibited that DKO-1-Exos had significantly upregulated IL-8 expression in neutrophils compared to DKs-8-Exos (Fig. 5c). It was worth noting that the protein expression of IL-8 was decreased by treatment with NETs inhibitor, DNAse, and the addition of exogenous IL-8 increased the IL-8 content in the medium, but the expression of IL-8 was decreased after the addition of anti-IL-8 (Fig. 5c).

We further evaluated the effect of IL-8 on the formation of NETs. We observed the formation of NETs by confocal microscopy and found that NETs formation was increased by addition of IL-8 or DKO-1-Exos in PMA-stimulated neutrophils compared with PBS treatment only. Combined treatment of IL-8 and DKO-1-Exos further increased NETs formation compared to treatment with either IL-8 or DKO-1-Exos alone. In contrast, anti-IL-8 treatment reversed the NETs formation induced by DKO-1-Exos (Fig. 5 d).

The aforementioned results revealed that the formation of NETs could be induced by mutant KRAS exosomes through the upregulation of IL-8.

### Mutant KRAS exosomes promote the NET formation and lead to CRC deterioration

**Fig. 6:**
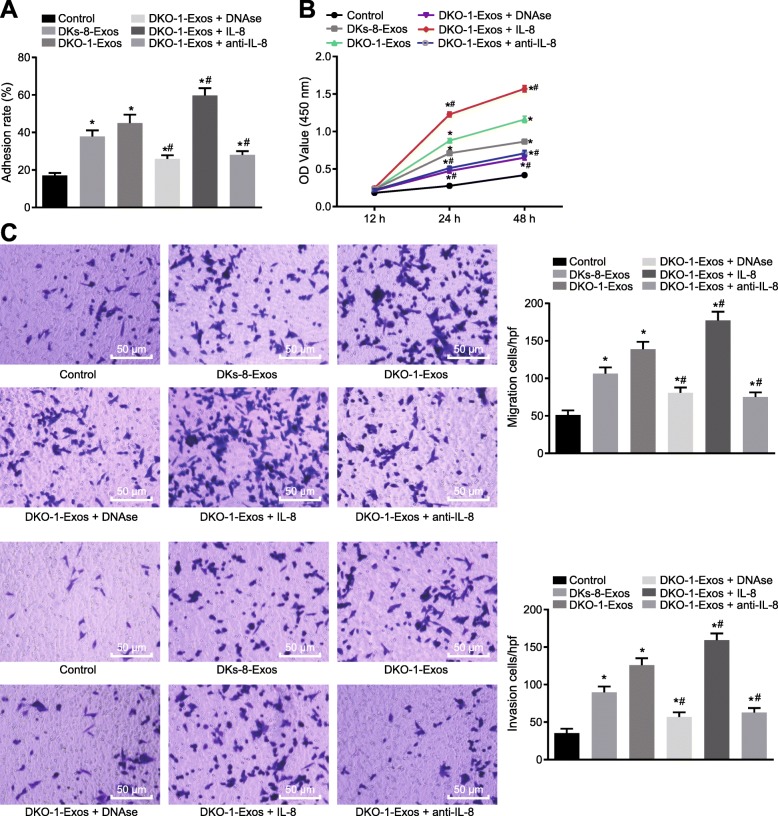
Mutant KRAS exosomes promote the NET formation and lead to CRC deterioration; **a**, The adhesion ability of DKs-8 cells; **b**, The viability of DKs-8 cells detected by CCK-8; **c**, The invasion and migration ability of DKs-8 cells assessed by Transwell assay (200×); * *p* < 0.05 compared with the control cells, and # *p* < 0.05 compared with DKO-1-Exos. The measurement data was expressed as mean ± standard deviation. Data comparisons between groups were performed using one-way analysis of variance, followed by Tukey’s post hoc test. Data analysis at different time points was performed by repeated measurement ANOVA, followed by Bonferroni post hoc test. The experiment repeated three times

To investigate the effect of mutant KRAS-mediated NETs formation on cancer cell growth in vitro, adhesion assay was conducted, As shown in Fig. 6a, the adhesion ability of the DKs-8 cells was improved by treatment with either DKs-8-Exos or DKO-1-Exos (*p* < 0.05). However, the tumor adhesion ability enhanced by DKO-1-Exos was significantly reduced after treatment with DNAse which inhibited the formation of NETs, and DKs-8 tumor adhesion induced in the presence of DKO-1-Exos was significantly increased after the addition of IL-8, but decreased after addition of anti-IL-8 (Fig. 6a, *p* < 0.05). Next, we assessed the direct effect of NETs on the proliferation of DKs-8 cells by CCK8 assay. The results showed that DKO-1-Exos significantly enhanced the growth of DKs-8 cells in conditional medium collected after PMA stimulated the neutrophil monolayer. Addition of DNAse or anti-IL-8 reduced the accelerated growth of tumor cells induced by DKO-1-Exos (Fig. 6b). However, the rate of cell growth enhanced by DKO-1-Exos was further increased after addition of IL8 (Fig. 6b). In addition, we further examined the effects of neutrophils treated with DKO-1-Exos on invasion and migration of DKs-8 cells. The Transwell assay results (Fig. 6c) showed that the migration and invasion abilities of DKs-8 cells were enhanced by treatment with either DKs-8-Exos or DKO-1-Exos. Whereas, DNAse treatment reversed the promotive effect of DKO-1 Exos on migration and invasion abilities of DKs-8 cells. Importantly, IL-8 treatment was able to further accelerate DKO-1-Exos-induced invasion and migration, but the invasion and migration abilities of DKs-8 cells induced by DKO-1-Exos were decreased after addition of anti-IL-8. In summary, the upregulation of IL-8 by mutant KRAS exosomes could stimulate the formation of NETs and led to the deterioration of CRC.

## Discussion

CRC remains one of the most lethal malignant diseases particularly in developed countries. The mortality caused by this cancer is relatively high in late stage. Identifying more effective clinically prognostic markers for early stage of CRC is currently recommended by many doctors and scientists. One approach is to surveil the response of immune system to pathological changes in tissues, since tumor cells can reside in specific tissues and normally grow for a long time via immune-escape [19]. Considering the abundant human intestinal floras in human, studies have speculated on the relationship between CRC and localized inflammation. Inflammation may trigger the release of harmful produce reactive oxygen species (ROS), warning an impair happening in our body. And then cytokines such as IL-6, IL-8 and IL-17 are produced to help epithelial growth and angiogenesis locally, which have been identified in the development of CRC as a result of infection with pathogenic bacteria in mice [20, 21]. However, the mechanism by which the CRC cells regulates the immunocytes to help tumor growth through chemokines is still unclear. Therefore, the current study aimed to investigate the role by which IL-8 influences KRAS-mutant CRC. Our results demonstrated that CRC cells transferred mutant KRAS protein to neutrophil and elevated formation of NETs increased secretion of IL-8 and in vivo as well as promoted proliferation of cancer cells in vitro.

A key finding of the current study revealed the upregulation of IL-8 and increased formation of NETs in KRAS-mutant CRC tissue. Interleukin-8 is a chemokine and can be produced by any cells with toll-like receptors such as macrophages, epithelial cells and endothelial cells [22]. IL-8 induces chemotaxis and guides target cells, primarily neutrophils, to congregate at the site of infection. Many other types of human cancers also promoted IL-8, including prostate cancer, nasopharyngeal carcinoma and hepatocellular carcinoma in addition to CRC [23]. Specially, IL-8 serum level in patients with ovarian carcinoma was significantly decreased during or after paclitaxel-containing chemotherapy, implying IL-8 as a promising indicator for cancer therapy. NETs are extracellular DNA fiber nets produced by neutrophils, which are generally used to catch pathogens like bacteria, fungal cells or viruses [24]. Patients with CRC exhibit increased release of NETs [17]. Besides, inflammatory molecules such as neutrophils and IL-8 are considered to play a key role in the formation of NET, and the long-term exposure to NET-related cascade reaction is associated with autoimmune diseases, which may cause damage on systemic organs [25]. Additionally, in non-small cell lung cancer, the oncogenic KRAS can induce the IL-8 overexpression that is directly associated with the production of NETs [18, 26]. Our results provided evidence suggesting that the formation of NETs was induced by KRAS-mutant cancer-derived exosomes through enhancement of IL-8. Similarly, other interleukin members have been implicated in NETs formation. For instance, IL-1β have been shown to be co-localized with NETs in the detected human abdominal aortic aneurysms (AAAs) and in vitro tests have revealed that IL-1β induces the formation of NETs which is specifically inhibited by IL-1RA [27]. As previous study reported, IL-8 may bind to IL-8-R1/2 in the neutrophil membrane to shuttle neutrophils to tumors [28, 29]. Meanwhile, IL-8 could promote the formation of NET by activating Src, ERK and p38 signaling [30]. Nevertheless, the molecular mechanism of IL-8 function on regulating formation of NETs is lack of details, which should be paid more attention in the following experimentation.

Furthermore, we also demonstrated that NETs formation in neutrophils not only elevated proliferation of cancer cells but also promoted their ability of metastasis and invasion. NETs are a defense against invading microbes as well as carcinomas cells. However, accumulating studies have offered data suggesting the opposite effects exerted by NETs, a finding of which was consistent with the results of the current study. For example, in a PAD4-deficient model, G-CSF release in blood could promote formation of NETs subcutaneously in Lewis lung carcinoma tumor. In pancreatic cancer, extracellular DNA caused by inflammation was strongly attributed to elevated invasion and metastasis of pancreatic cancer cells [31]. In regard to lung cancer, NETs have been found to capture circulating tumor cells and boost them to migrate to liver significantly [32]. Likewise, we demonstrated that growth, invasion and migration of CRC cells were significantly elevated after co-culture with DKO-1-Exos, which were reversed by DNase treatment. However, the exact molecular mechanism that NETs functions to regulate tumor growth and transition remains unclear. The reasonable speculation was that DNA fibers in NETs provided contacted stroma to capture cancer cells, which might activate the epithelial signaling pathway and promote the cellular ability of proliferation and metastasis. Another hypothesis was that DNA fiber in NETs twined around cancer cells and facilitated them to park in certain locus along blood vessel. However, all reported experiments were carried out in vitro. More elaborated and in vivo experiments should be arranged in future.

## Conclusion

**Fig. 7:**
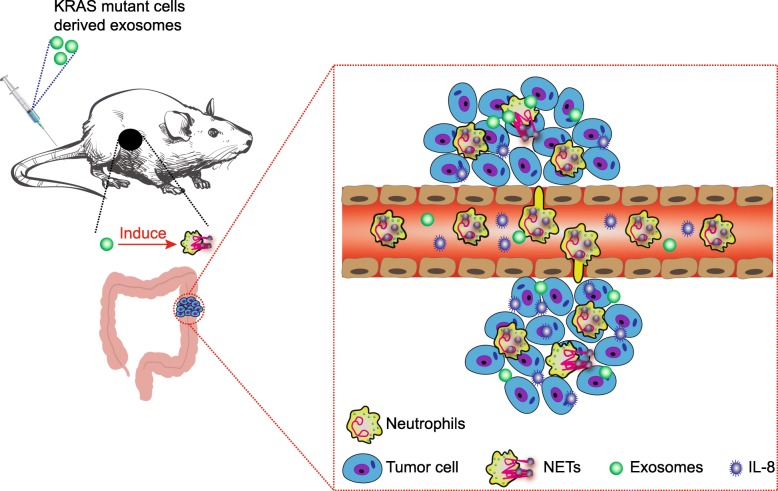
Schematic diagram of exosomal KRAS mutation implicated in the tumor microenvironment of colorectal cancer. Exosomes transfer mutant KRAS to recipient cells and increase IL-8 production, promote neutrophil recruitment and form NETs, eventually leading to the deterioration of CRC in murine models

In conclusion, the key findings of the present study identified IL-8 upregulation and neutrophils enrichment in KRAS-mutant CRC tissues. Exosomes transfer mutant KRAS to recipient cells and increase IL-8 production, promote neutrophil recruitment and form NETs, which ultimately leads to the deterioration of CRC (Fig. 7). This study provides insight into the relationship between mutant KRAS-containing exosomes and the development of CRC. Neutrophils as well as many other immunocytes may possess dual characteristics towards tumor cells, which could be potentially used by tumor cells to cross over the human immune system. Hence, further investigation is required in order to elucidate the finer details regarding the physiological function of neutrophils. Besides, the specific cell types in which KRAS mutation activates IL-8 in CRC remain to be further explored.

## Data Availability

The datasets used and/or analysed during the current study are available from the corresponding author on reasonable request.

## Abbreviations

AAAs: Abdominal aortic aneurysms
ACUC: Animal Care and Use Committee
ANOVA: Analysis of variance
APC-WT: APC with wild-type Kras
BM: Bone marrow
CAC: Colitis-associated cancer
CCK-8: Cell counting kit-8
CRC: Colorectal cancer
DMEM: Dulbecco’s Modified Eagle Medium
ECL: Enhanced chemiluminescence
ELISA: Enzyme-linked immunosorbent assay
FBS: Fetal bovine serum
IBD: Inflammatory bowel disease
IL-8: Interlukin-8
LC-MRM: LC-multiple reaction monitoring
mLN: Mesenteric lymph nodes
MPO: Myeloperoxidase
NET: Neutrophil extracellular trap
NETs: Neutrophil extracellular traps
OD: Optical density
PBS: Phosphate-buffered saline
PMN: Human peripheral blood neutrophils
ROS: Reactive oxygen species
